# Synthetic Approaches to the Lamellarins—A Comprehensive Review

**DOI:** 10.3390/md12126142

**Published:** 2014-12-18

**Authors:** Dennis Imbri, Johannes Tauber, Till Opatz

**Affiliations:** Institute of Organic Chemistry, Johannes Gutenberg-University Mainz, Duesbergweg 10-14, 55128 Mainz, Germany; E-Mails: d.imbri@uni-mainz.de (D.I.); tauberj@uni-mainz.de (J.T.)

**Keywords:** lamellarins, pyrroles, alkaloids, marine natural products, total synthesis

## Abstract

The present review discusses the known synthetic routes to the lamellarin alkaloids published until 2014. It begins with syntheses of the structurally simpler type-II lamellarins and then focuses on the larger class of the 5,6-saturated and -unsaturated type-I lamellarins. The syntheses are grouped by the strategy employed for the assembly of the central pyrrole ring.

## 1. Introduction

**Figure 1:**
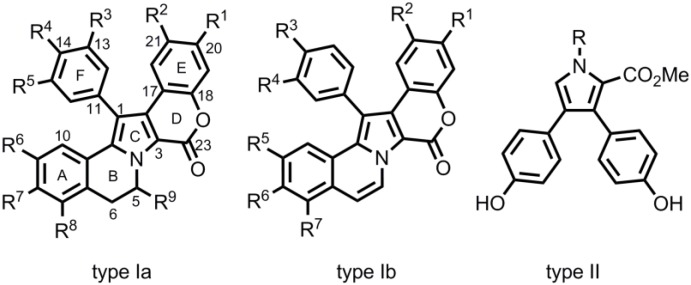
Structure of lamellarins type Ia, Ib and II [16].

The Lamellarins were first isolated by Faulkner and co-workers in 1985 from the prosobranch mollusc *Lamellaria* sp. [1,2,3,4,5,6,7,8,9,10,11] and represent a group of more than 50 polycyclic marine alkaloids which contain a central pyrrole moiety. The pyrrole ring may be part of a fused pentacyclic skeleton of the 6*H*-chromeno[4′,3′:4,5]pyrrolo[2,1-*a*]isoquinolin-6-one type possessing a partially saturated (type Ia) or fully unsaturated (type Ib) isoquinoline moiety [12,13,14,15]. Furthermore, structurally simpler representatives of the lamellarins containing a 3,4-diaryl substituted pyrrole core (type II) are known (Figure 1) [4,5]. Their substitution in terms of a varying oxygenation and O-substitution pattern provides the basis for their structural diversity as well as for notable differences in their biological activity.

Many lamellarins were found to exhibit significant biological effects on mammalian cells or even viruses, such as antiproliferative and multidrug resistance reversal activity, cytotoxicity, or the inhibition of HIV-1 integrase [17,18,19,20,21,22,23]. Structure activity relationships were investigated with a particular emphasis on lamellarin D, the most cytotoxic representative of the lamellarins [24,25,26,27,28]. These studies resulted in a pharmacophore model explaining the structural features required for inhibiting the action of eukaryotic topoisomerase I, an enzyme required for transcription and replication of DNA [29,30,31,32,33,34,35,36]. In order to provide samples of the natural lamellarins or their synthetic derivatives for biomedical research [37,38,39,40,41], various synthetic strategies have been developed. While synthetic, biological and biomedical as well as biophysical aspects of the lamellarins have already been the subject of various reviews [42,43,44,45,46,47,48,49,50,51,52,53,54,55,56,57,58,59,60,61,62,63,64], we herein present an overview of all synthetic approaches published until today (2014). The syntheses are grouped by the class of the lamellarin synthesized (type II *vs.* type Ia/Ib) as well as by the strategy used for the construction and/or decoration of the central pyrrole ring.

## 2. Syntheses of type-II Lamellarins

### 2.1. Fürstner 1995 [65]

**Scheme 1:**
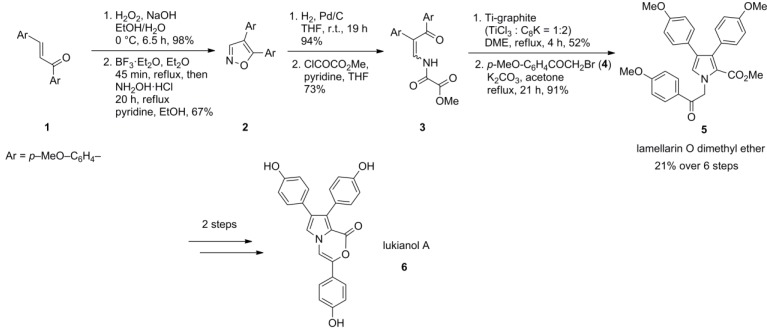
Synthesis of lamellarin O dimethyl ether by Fürstner *et al.* [65].

A decade after the discovery of the lamellarins [1] Fürstner and co-workers reported in 1995 the first total synthesis of a type-II lamellarin [65] characterized by a non-fused pyrrole moiety. In his approach to lukinaol A (**6**), lamellarin O dimethyl ether (**5**) was used as an intermediate. The route started with a Scheffer-Weitz-epoxidation of enone **1** followed by Lewis acid-mediated rearrangement and condensation with hydroxylamine to afford isoxazole **2**. Upon reductive cleavage of the N-O bond, condensation with methyl oxalyl chloride and McMurry-type cyclization with Ti-graphite, the 3,4,5‑trisubstituted pyrrole was formed which readily yielded lamellarin O dimethyl ether (**5**) upon N‑alkylation with 2-bromo-4′-methoxyacetophenone (**4**) in 21% over six steps (Scheme 1).

This and any further mentioned yields as well as the numbers of synthetic steps given in this review will, if not stated otherwise, refer to the respective data of the longest linear sequence as provided by the authors themselves. In case of convergent syntheses with a shorter but lower-yielding branch, the overall yield stated refers to the yield-limiting sequence.

### 2.2. Banwell 1997 [66]

**Scheme 2:**
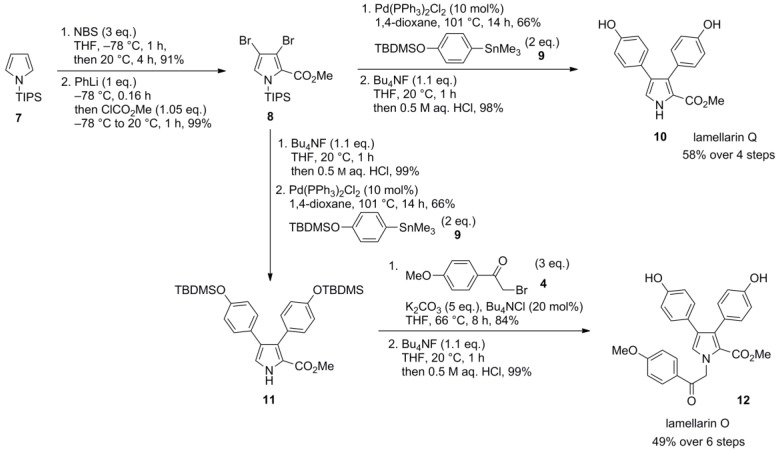
Synthesis of lamellarins O and Q by Banwell *et al.* [66].

Cross-couplings such as Stille, Suzuki or Negishi reactions served as the key steps in the synthesis of lamellarin O, lamellarin Q and lukianol A by Banwell and co-workers. The TIPS-protected pyrrole **7** was tribrominated with three equivalents of *N*-bromosuccinimide to form the tribromo derivative which was subjected to halogen-lithium exchange with phenyllithium followed by quenching with methyl chloroformate to afford dibromopyrrole 2-carboxylate **8**. Lamellarin Q (**10**) was then synthesized by twofold Stille cross-coupling with aryl stannane **9** and subsequent desilylation with Bu_4_NF in 58% over four steps starting from **7**. To prevent O*-*alkylation, the synthesis of lamellarin O was initiated with an N*-*desilylation followed by twofold Stille cross-coupling with aryl stannane **9**. The coupling product **11** was subjected to N*-*alkylation with 2-bromo-4′-methoxyacetophenone (**4**) followed by desilylation with Bu_4_NF to provide lamellarin O (**12**) in 45% over six steps. The authors reported that no significant quantities of mono-arylated pyrroles were formed even in the case of shorter reaction times or equimolar amounts of the coupling partner. Hence, differentially diarylated pyrroles were not readily accessible by this approach (Scheme 2).

Banwell and co-workers demonstrated a method to overcome this limitation by regioselective halogen-lithium exchange and subsequent transmetallation into an organozinc derivative, thus allowing the differentiation between positions 3 and 4 in the pyrrole unit.

### 2.3. Boger 1999 [67]

**Scheme 3:**
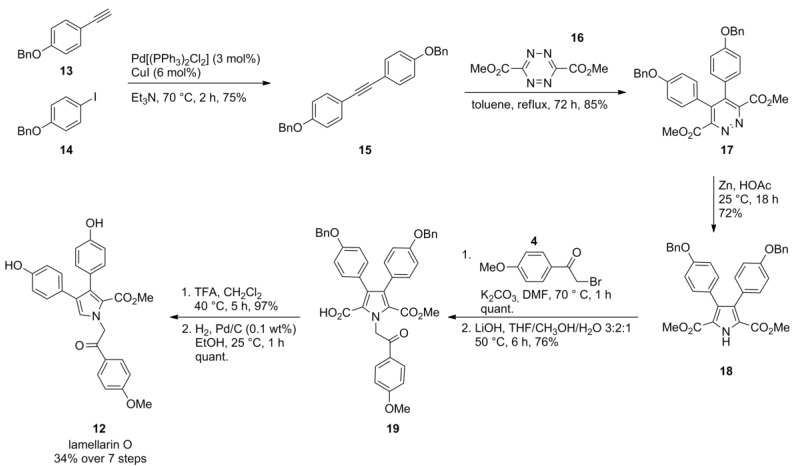
Synthesis of lamellarin O by Boger *et al.* [67].

An azadiene Diels-Alder strategy, namely a 1,2,4,5-tetrazine-1,2-diazine-pyrrole transformation sequence, was used by Boger and co-workers for the construction of the lamellarin framework. The pyrrole moiety was thus assembled by a [4+2] cycloaddition/cycloreversion reaction followed by a reductive ring transformation, a method infrequently used for the construction of five membered heteroaromatic systems. Starting from benzyl protected aryl acetylene **13** and aryl iodide **14,** the acetylenic precursor **15** was prepared by Sonogashira-coupling. The undesired homocoupling of the alkyne component was suppressed by its slow addition to a solution of the iodide **14**. Cycloaddition/cycloreversion of tolan **15** to 1,2,4,5-tetrazine **16** gave 1,2-diazine **17** which was subjected to zinc-mediated reductive ring contraction followed by N*-*alkylation with 2-bromo-4′-methoxyacetophenone (**4**). Selective hydrolysis of the symmetrical diester **18** with lithium hydroxide provided the monoacid **19** which was decarboxylated in the presence of an excess of trifluoroacetic acid. Subsequent hydrogenolysis afforded lamellarin O (**12**) in 34% overall yield over seven steps (Scheme 3).

### 2.4. Álvarez 2004 [68]

A solid phase synthesis of lamellarin Q and O was reported by Álvarez and co-workers. They employed a variation of Banwell’s route of 1997 (*vide supra*) [66] which was adapted to the requirements of solid phase organic chemistry. Different strategies of linking the central pyrrole unit to the resin were investigated while linking through the nitrogen was ruled out due to the lacking flexibility in introducing diversity at this position.

**Scheme 4:**
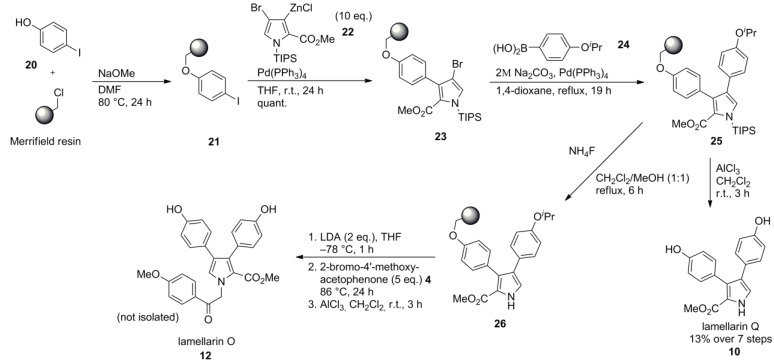
Synthesis of lamellarins O and Q by Álvarez *et al.* [68].

The resin-bound pyrrole **23** was obtained by Negishi cross-coupling of the highly functionalized organozinc reagent **22**, the preparation and utility of which had been demonstrated earlier by Banwell, with the resin-bound iodophenol **21**. A quantitative coupling yield was ensured by using a tenfold excess of the haloarene. Two possible routes, either with the zinc attached to the immobilized reactant or to the soluble coupling partner, were taken into account. While the zinc derivative of resin-bound pyrrole failed to meet the expectations, Suzuki-Miyaura cross-coupling reaction of **23** with boronic acid **24** provided the corresponding diarylpyrrole** 25**. Treatment with AlCl_3_ induced a simultaneous N*‑* and O*-*deprotection as well as the cleavage from the resin, yielding lamellarin Q (**10**) in 13% yield. On the other hand, N-desilylation with NH_4_F followed by N-alkylation of **26** with 2‑bromo‑4′‑methoxyacetophenone (**4**) and subsequent O*-*deprotection/cleavage with AlCl_3_ provided lamellarins O (**12**) and Q (**10**) as a mixture which proved to be impossible to separate by HPLC (Scheme 4).

### 2.5. Iwao 2008 [69]

**Scheme 5:**
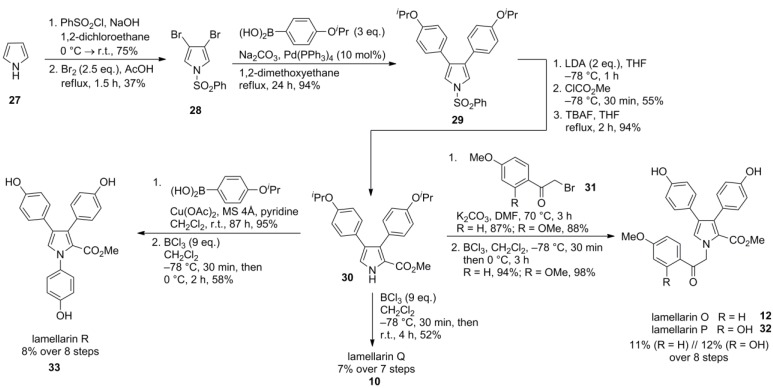
Synthesis of lamellarins O, P, Q and R by Iwao *et al.* [69].

The Suzuki-Miyaura cross-coupling approach used by Banwell in 1997 (*vide supra*) [66] was refined by Iwao and co-workers in the synthesis of lamellarins O, P, Q, and R. Therefore, *N*‑benzenesulfonyl-3,4-dibromopyrrole (**28**) was subjected to twofold Suzuki-Miyaura cross-coupling providing the diarylpyrrole **29**. The dibromopyrrole intermediate **28** is readily available from pyrrole (**27**) by sulfonylation and bromination with Br_2_ in acetic acid in 28% over two steps. The kinetically disfavored β‑bromination becomes practicable under thermodynamic conditions in acidic medium. In contrast to the Banwell route, the ester moiety was introduced on a later stage by lithiating diarylpyrrole **29** with LDA and subsequent quenching with methyl chloroformate. Upon N*‑*deprotection with Bu_4_NF, pyrrole **30** was obtained which was further deprotected with BCl_3_ (Iwao had demonstrated already in his synthesis of 2005 that BCl_3_ is a selective reagent for the cleavage of benzyl or isopropyl ethers in lamellarin precursors without affecting methyl ethers, which on the other hand are easily removed by BBr_3_—*vide infra*) to afford lamellarin Q (**10**) in 7% over seven steps (25% from dibromopyrrole **28**). N-Alkylation of **30** with the corresponding bromide **31** followed by cleavage of the isopropyl ethers with BCl_3_ afforded lamellarin O (**12**) and P (**32**) in 11% and 12% over eight steps, respectively (40% and 43% from **28**). Lamellarin R (**33**) was synthesized by a Chan-Lam-Evans-type N*-*arylation of **30** followed by O-deprotection with BCl_3_ in 8% over eight steps (29% from **28**, Scheme 5).

### 2.6. Jia 2011 [70]

Keeping the structural properties of the lamellarins in mind and considering the differences between type I and type II lamellarins, it appears reasonable to assume that the type II structures precede those of type I in the biosynthesis. In this context, Jia and co-workers reported a biomimetic approach comprising three oxidative ring closures, reactions that are commonly seen in biosynthetic pathways towards densely functionalized secondary metabolites.

**Scheme 6:**
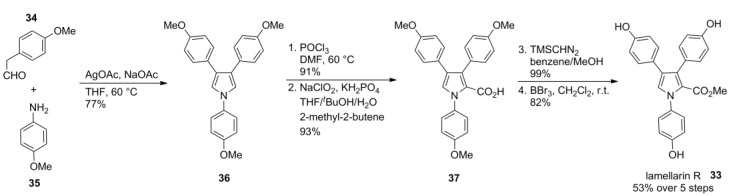
Synthesis of lamellarin R by Jia *et al.* [70].

Starting from *p*-methoxyphenylacetaldehyde (**34**) and *p*-anisidine (**35**), AgOAc-mediated oxidative coupling afforded pyrrole **36** in the key step of Jia’s elegant synthesis. Vilsmeier-Haack formylation followed by Pinnick/Lindgren oxidation (under the optimized conditions discussed in 5.4) provided carboxylic acid **37** which was methylated with TMSCHN_2_ to afford lamellarin R (**33**) after a threefold O-demethylation with BBr_3_ in 53% over five steps (Scheme 6).

### 2.7. Vazquez 2012 [71]

**Scheme 7:**
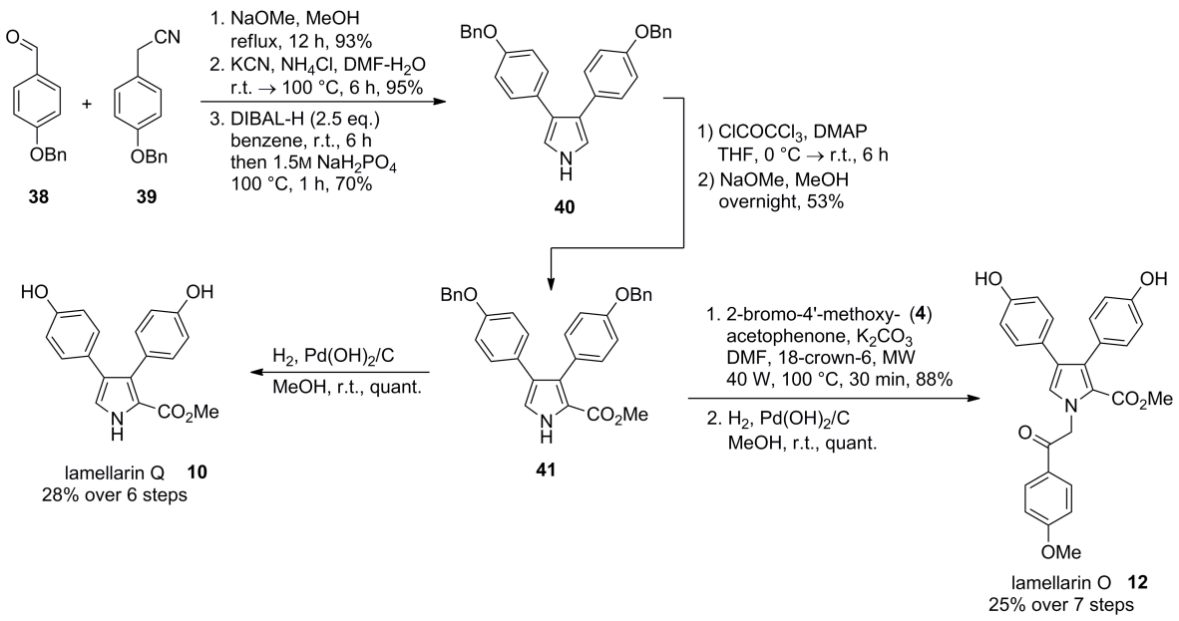
Synthesis of lamellarins O and Q by Vazquez *et al.* [71].

The Paal-Knorr pyrrole synthesis has been chosen by Vazquez and co-workers as an alternative key step for their synthesis of lamellarins O (**12**) and Q (**10**). Here, a 2,3-diarylsuccinonitrile was used as a 1,4-dicarbonyl analogue. The approach is based on the Knoevenagel condensation between aldehyde **38** and arylacetonitrile **39** followed by conjugate addition of cyanide to the resulting cyanostilbene. Upon reduction with DIBAL-H the dialdehyde was formed *in situ* and cyclized to the diarylpyrrole **40** under slightly acidic pH by addition of sodium dihydrogen phosphate. Introduction of the ester moiety by trichloroacetyl chloride failed. However, addition of Steglich’s 4-dimethylaminopyridine circumvented this problem. The methyl ester **41** resulted from methanolysis of the trichloroacetylated compound. Direct hydrogenolysis of the benzyl ethers afforded lamellarin Q (**10**) in 28% over six steps, whereas preliminary N*-*alkylation with 2-bromo-4′-methoxyacetophenone (**4**) followed by hydrogenolysis provided lamellarin O (**12**) in 25% over seven steps. The use of benzyl ethers remediated the need for strong Lewis-acids for O*-*protection (Scheme 7).

## 3. Syntheses of Type I-Lamellarins—Approaches via Halogenation and Cross-Coupling Reactions

### 3.1. Iwao 2003 (lamellarin G trimethyl ether) [72]-2006 (lamellarin D, L and N) [73]-2006 (lamellarin α 20‑sulfate) [74]-2010 (lamellarin α 20-sulfate, α 13-sulfate and α 13,20-disulfate) [75]

The strategy of using cross-coupling reactions to form the diarylpyrrole moiety for type II lamellarins was further investigated by Iwao and co-workers to allow the synthesis of the more complex type I-lamellarins. Commonly, lamellarin G trimethyl ether serves as a benchmark compound for comparing the efficiency of syntheses of the lamellarins as it obviates any protecting group chemistry and lacks the structural differentiation of the E- and F-ring precursor.

**Scheme 8:**
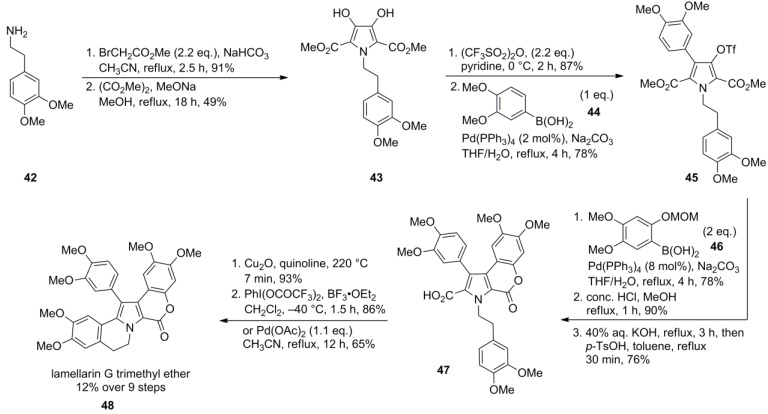
Synthesis of lamellarin G trimethyl ether by Iwao *et al.* [72].

For lamellarin G trimethyl ether (**48**), the synthesis started with commercially available homoveratrylamine (**42**). N-dialkylation with methyl bromoacetate followed by Hinsberg pyrrole cyclization with dimethyl oxalate provided the pentasubstituted pyrrole **43** (Scheme 8). Suzuki-Miyaura cross-coupling of the bistriflate derived from **43** to an arylboronic acid **44** (2.2 to 3.0 equiv.) afforded the diarylated pyrrole, whereas a stoichiometric amount of the arylboronic acid **44** primarily produced the monoarylated pyrrole **45**. Therefore, the sequential introduction of different arylboronic acids was feasible, a necessity for the formation of type I-lamellarins with respect to the regioselective construction of the D-ring lactone, *i.e.*, the discrimination of the rings E and F. Thus, one of the arylboronic acids (**46**) incorporated a MOM-protected phenolic function in *ortho* position to achieve lactonization upon acidic deprotection. To a small extent, lactone **47** was already formed as a by-product of the cross-coupling reaction. Saponification of the second methyl ester followed by Cu_2_O-catalyzed decarboxylation afforded the per-O*-*methylated ningalin B. Formation of the dihydroisoquinoline moiety (AB unit) was effected with a hypervalent iodine reagent under Kita’s biaryl coupling conditions (PIFA/BF_3_∙Et_2_O) [76]. Lamellarin G trimethyl ether (**48**) was obtained in 12% yield over nine steps.

The synthesis could be shortened by applying a Pd(II)‑mediated decarboxylative cyclization on monoacid **47**, a method inspired by the Pd(0)‑mediated decarboxylative cyclization of the analogous bromoacid **159** described by Steglich and co-workers in a previous article [77]. In this case, lamellarin G trimethyl ether (**48**) was obtained in 10% yield over eight steps. The ring closure via the Pd(II) salt proceeded regioselectively, presumably due to steric hindrance. As a side reaction, the unproductive decarboxylation of the pyrrole was observed (12%).

**Scheme 9:**
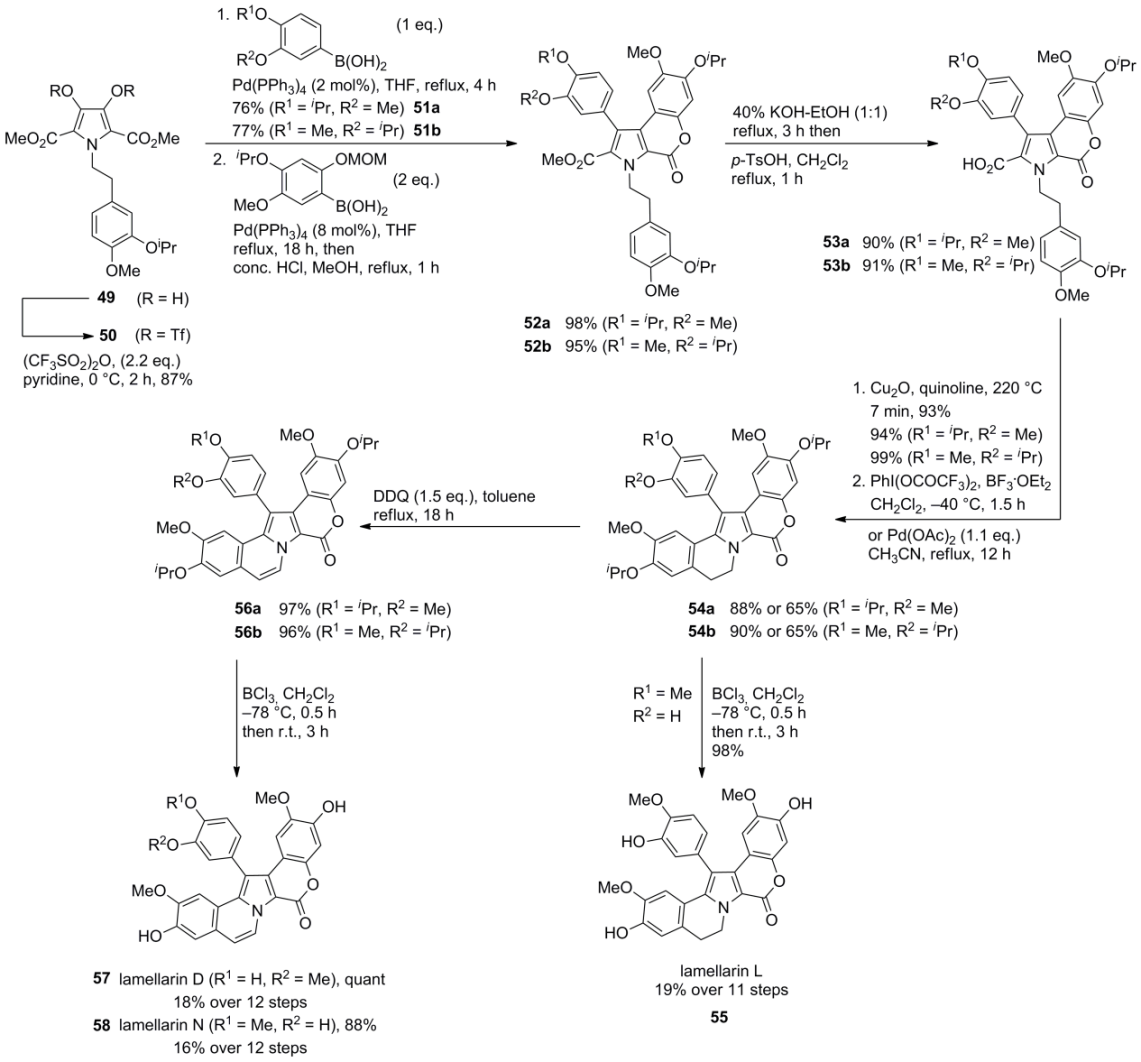
Synthesis of lamellarins D, N and L by Iwao *et al.* [73].

In contrast, predefining the hydroxy-methoxy-substitution pattern of the A-subunit, as found in lamellarins D (**57**), L (**55**) and N (**58**), was achieved by their introduction as a 3,4-dialkoxy-substituted aryl aldehyde. Starting from isopropyl-protected isovanillin, a sequence comprising nitroaldol condensation with CH_3_NO_2_, LiAlH_4_-reduction, N-dialkylation with methyl bromoacetate and Hinsberg pyrrole cyclization with dimethyl oxalate provided the common pentasubstituted pyrrole **49** (Scheme 9). Double O-triflylation with Tf_2_O furnished the pivotal bistriflate **50** in 32% over five steps.

Along these lines, the target compounds were obtained in total yields of 18% (lamellarin D, 12 steps), 19% (lamellarin L, 11 steps) and 16% (lamellarin N, 12 steps), respectively. Similar results as for the synthesis of lamellarin G trimethyl ether (**48**) were encountered in the alternative construction of the AB-subunit by Pd(II)‑mediated decarboxylative cyclization with slightly lower yields (14%, 14%, and 12%, respectively) and the concomitant formation of the decarboxylated by-products (14% for lamellarin D-precursor, 15% for lamellarin N*-*precursor, Scheme 9).

**Scheme 10:**
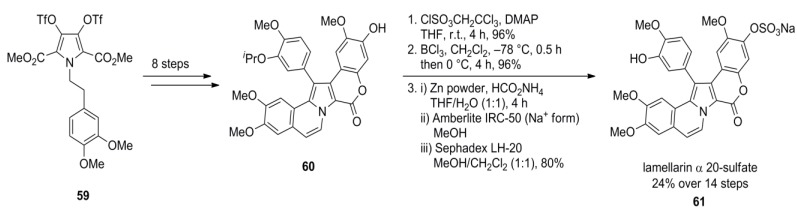
Synthesis of lamellarin α 20-sulfate by Iwao *et al.* [74].

**Scheme 11:**
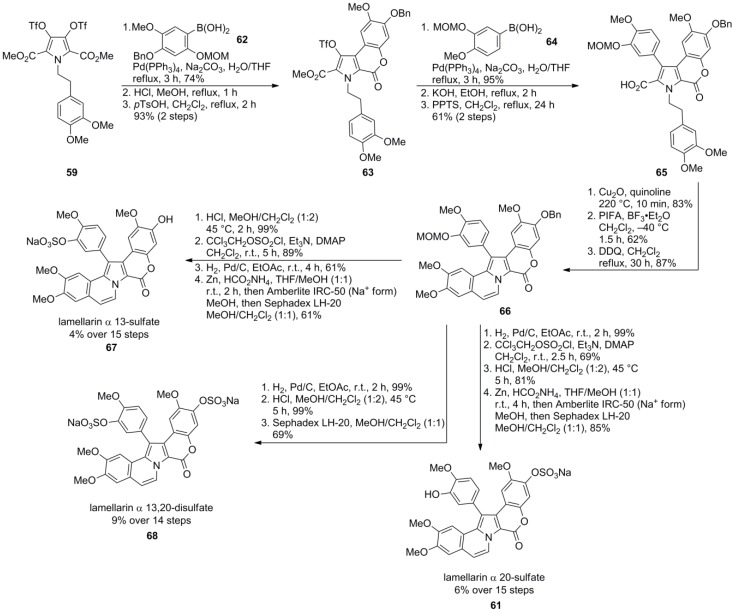
Synthesis of lamellarins α 13-sulfate, α 20-sulfate and α 13,20-disulfate by Iwao *et al.* [75].

In 2006, Iwao and co-workers extended their protocol by employing differentially protected aryl subunits E and F (isopropyl ether on F, benzyl ether on E) to obtain the selectively mono-protected pyrrole **60 **in 32% over eleven steps, starting from homoveratrylamine (**42**). Thereby, diverse sulfated lamellarins should be available for structure-activity relationship studies. Treatment with trichloroethyl chlorosulfate followed by isopropyl ether cleavage with BCl_3_ provided the trichloroethyl sulfate. Finally, reductive deprotection with Zn/ammonium formate and subsequent ion exchange (Amberlite IRC-50, Na^+^-form) gave lamellarin α 20-sulfate (**61**) in 24% over 14 steps (Scheme 10). Analogously, lamellarin α 13-sulfate (**67**) and lamellarin α 13,20-disulfate (**68**) were successfully synthesized from common differentially protected intermediate **66** by Iwao’s group in 5%, 6% and 9% yield over 15, 15, and 14 steps, respectively (starting from **42**). In contrast to their previous work, MOM-protection instead of the isopropyl ethers was used in combination with a different order of the Suzuki cross‑couplings and closure of the lactone ring prior to the second arylation (Scheme 11).

### 3.2. Handy 2004 [78]

The first total synthesis of type I-lamellarins starting from pyrrole itself was reported by Handy and co-workers in 2004. A triply iterative halogenation/cross-coupling reaction sequence was applied in a regioselective fashion to obtain lamellarin G trimethyl ether as the benchmark compound for a modular synthesis of natural lamellarins.

**Scheme 12:**
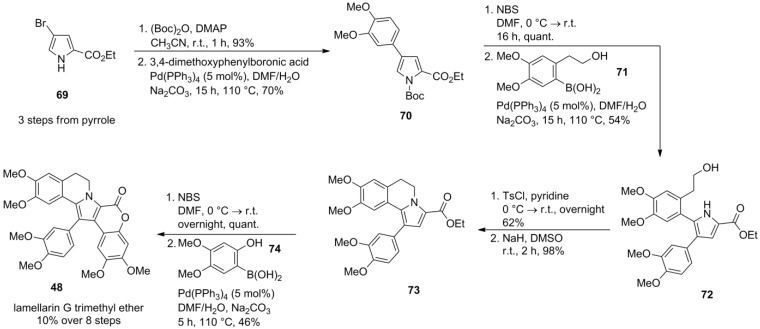
Synthesis of lamellarin G trimethyl ether by Handy *et al*. [78].

Initially, bromopyrrole ester **69** (prepared in three steps from pyrrole, no yield stated) was N-protected with a Boc-group prior to Suzuki-Miyaura coupling with 3,4-dimethoxyphenylboronic acid to avoid extensive dehalogenation. A modest excess (2–3 equiv.) of the boronic acid was required to achieve full conversion. Regioselective bromination with NBS at the more reactive 5-position of pyrrole **70** followed by a second Suzuki coupling with arylboronic acid **71** provided pyrrole **72**, while no protection of the free hydroxyl group was necessary. The dihydroisoquinoline moiety was established by O*-*tosylation of the primary alcohol and subsequent intramolecular N*-*alkylation. A final bromination followed by a third Suzuki coupling with arylboronic acid **74** and simultaneous lactonization afforded lamellarin G trimethyl ether (**48**) in 10% over eight steps starting from pyrrole **69**. It is noteworthy that the final cross-coupling required extensive optimization due to low initial yields, and an improvement from 8% to 46% in that particular step could be achieved although a large excess of boronic acid was necessary (8 equiv.) and the main product (51%) still was the dehalogenated compound **73** (Scheme 12).

### 3.3. Álvarez 2005 [79]

Lamellarin D was synthesized by Álvarez and co-workers starting from pyrrole ester **75** (available in two steps from pyrrole itself), thus presenting a generally applicable synthesis for other lamellarins, as lamellarin D already requires a regiodifferentiation strategy due to its unsymmetrical hydroxy/methoxy substitution pattern.

**Scheme 13:**
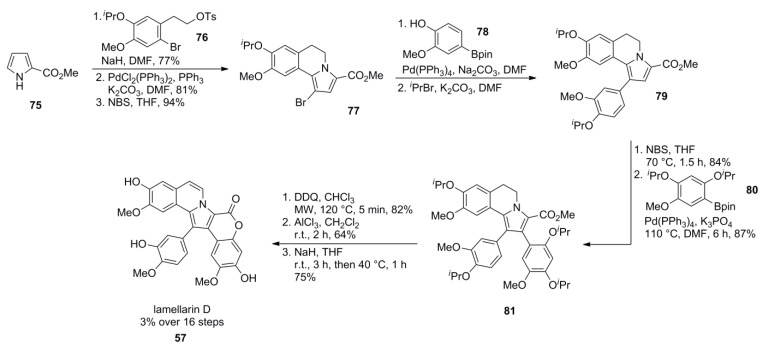
Synthesis of lamellarin D by Álvarez *et al*. [79].

The AB subunit of the lamellarins was established by N*-*alkylation of pyrrole ester **75** with tosylate **76** which itself was prepared in a six step reaction sequence including Baeyer-Villiger oxidation, O*-*isopropylation, bromination, Wittig homologation, reduction and O*-*tosylation in 27% overall yield. Palladium-catalyzed Heck-type cyclization followed by a regioselective bromination provided tricyclic building block **77** which was subjected to Suzuki-Miyaura cross-coupling, subsequent O*-*isopropylation of the phenolic group and a second bromination-Suzuki cross-coupling sequence, as already described by Handy and co-workers [78]. The introduction of the aryl unit requires a large excess of boronic acid **80**, presumably due to its steric hindrance. Introduction of the 5,6-double bond was accomplished by DDQ oxidation. Finally, cleavage of the isopropyl ethers with AlCl_3_ followed by acidic lactonization afforded lamellarin D (**57**) in 3% over 16 steps (Scheme 13).

### 3.4. Banwell 2011 [80]

An entirely modular approach to the type I-lamellarins was reported by Banwell and co-workers. It consists of the successive attachment of prefunctionalized aryl units to the pyrrole moiety via Suzuki‑Miyaura cross-couplings and a decarboxylative Heck reaction to close the B-ring.

The construction of the regioselectively functionalized pyrrole **84** started from Boc-protected pyrrole **82** with a double bromination at C2 and C5, double halogen-lithium exchange and quenching with methyl chloroformate to afford diester **83**. Treatment of **83** with *N*-iodosuccinimide (2 equiv.) followed by zinc promoted desymmetrization provided iodopyrrole **84** which was subjected to Suzuki coupling with *ortho*-hydroxy boronate ester **85**, simultaneously forming the lactone subunit as already encountered in Iwao’s approach [72]. Thereafter, bromination with *N*-bromosuccinimide followed by N*-*alkylation under Mitsunobu conditions and Suzuki coupling with the corresponding boronate ester afforded pyrrole **88**. Saponification and treatment with *p*-toluenesulfonic acid gave the fused pyrrole carboxylate which underwent palladium-mediated cyclization under decarboxylation.

**Scheme 14:**
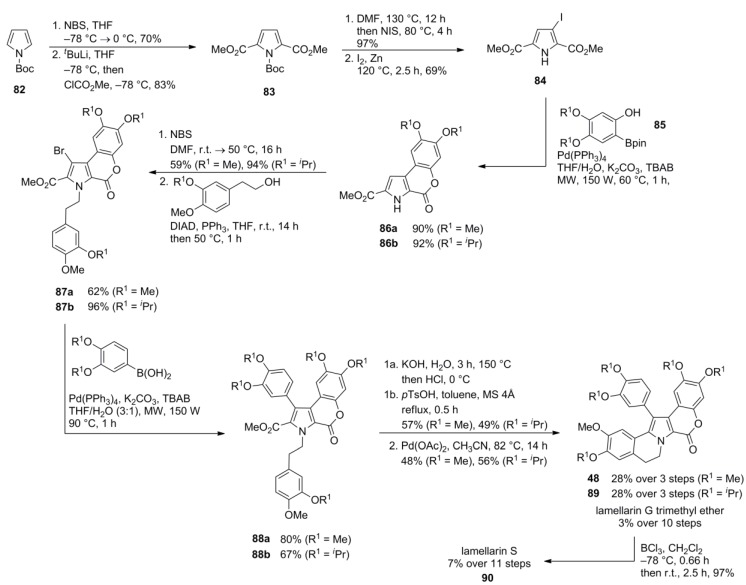
Synthesis of lamellarin G trimethyl ether and lamellarin S by Banwell *et al*. [80].

Accordingly, lamellarin G trimethyl ether (**48**) was obtained in 3% over 10 steps and general applicability was shown with the first total synthesis of lamellarin S (**90**) in 7% over 11 steps from 3,4‑diisopropoxybenzaldehyde. In the latter case, the isopropyl ethers were cleaved following the commonly used BCl_3_ protocol (Scheme 14).

### 3.5. Iwao 2014 [81]

**Scheme 15:**
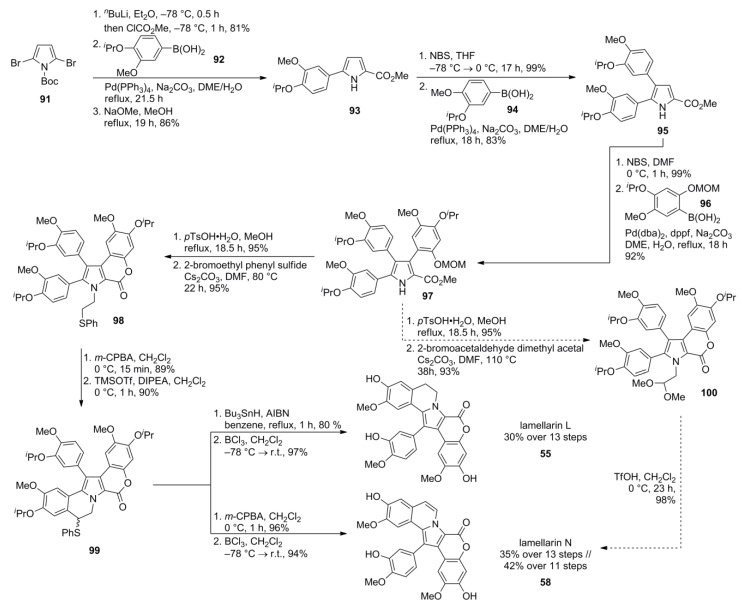
Synthesis of lamellarins N and L by Iwao *et al*. [81].

Iwao and co-workers recently developed a slightly different route from dibromopyrrole **91**, the intermediate also used in Banwell’s previously described strategy (*vide supra*) [80]. Instead of converting both bromine substituents into methoxycarbonyl units, the authors chose to convert only one, and attached the A-ring at an early stage, a connection done at a much later stage by Banwell and co-workers who attached the A-ring by closing the B-ring in the ultimate step (except deprotections). Suzuki-Miyaura cross-coupling with boronic acid **92** affords arylated pyrrole **93**. Bromination of **93** proceeded regioselectively as in the synthesis of Álvarez [79]. Suzuki coupling, final bromination at the remaining position of the pyrrole ring, followed by another Suzuki coupling (each with a different arylboronic acid) yielded the lactone precursor **97**. Acidic removal of the MOM group resulted in closure of the lactone ring. Two possible pathways for annulation of the B-ring via initial N*-*alkylation were attempted, with 2-bromoethyl phenyl sulfide (Pummerer route) or bromoacetaldehyde dimethyl acetal as two-carbon homologation agents. N*-*alkylation of the lactone with 2-bromoethyl phenyl sulfide followed by oxidation and TMSOTf mediated Pummerer cyclization (Craig-modification) provided phenylthio-lamellarin **99**, whereas the application of standard reaction conditions (excess of triflic anhydride) mainly led to the reduced precursor. Radical desulfurization of **99** with Bu_3_SnH/AIBN provided access to saturated lamellarins (type Ia), whereas oxidation with *m*-CPBA followed by elimination of the intermediate sulfoxide generated unsaturated lamellarins (type Ia). Hence, an additional oxidative step as hitherto realized by treatment with DDQ was not required. Alternatively, N*-*alkylation of the lactone with bromoacetaldehyde dimethyl acetal followed by acid catalyzed cyclization (TfOH) of acetal **100** afforded unsaturated lamellarins in a more convenient and shorter fashion. In summary, lamellarin L (**55**) and N (**58**) were obtained in 29% and 34% over 13 steps, respectively. Alternatively, lamellarin N was obtained in 42% yield over 11 steps using the acetal based annulation route (Scheme 15).

## 4. Syntheses of Type I-lamellarins—Approaches via N*-*Ylide-Mediated Pyrrole Ring Formation

### 4.1. Iwao 1997 [82]

Iwao and co-workers reported the earliest synthesis of the most cytotoxic and potent representative of the lamellarins, lamellarin D [24] as well as the fully demethylated lamellarin H by assembly of the pyrrole unit at a later stage. The required papaverine derivative **101** was obtained from *O*‑benzylisovanillin after a sequence of nitroaldol condensation, conjugated addition of methanol, LiAlH_4_-reduction, amide condensation and Bischler-Napieralski cyclization in 26% over five steps. The MOM‑protected benzoate **102** was obtained in 49% over four steps from methyl 2,4-dihydroxybenzoate by O*-*benzylation, bromination, Br/OMe exchange and protection with MOM-Cl.

**Scheme 16:**
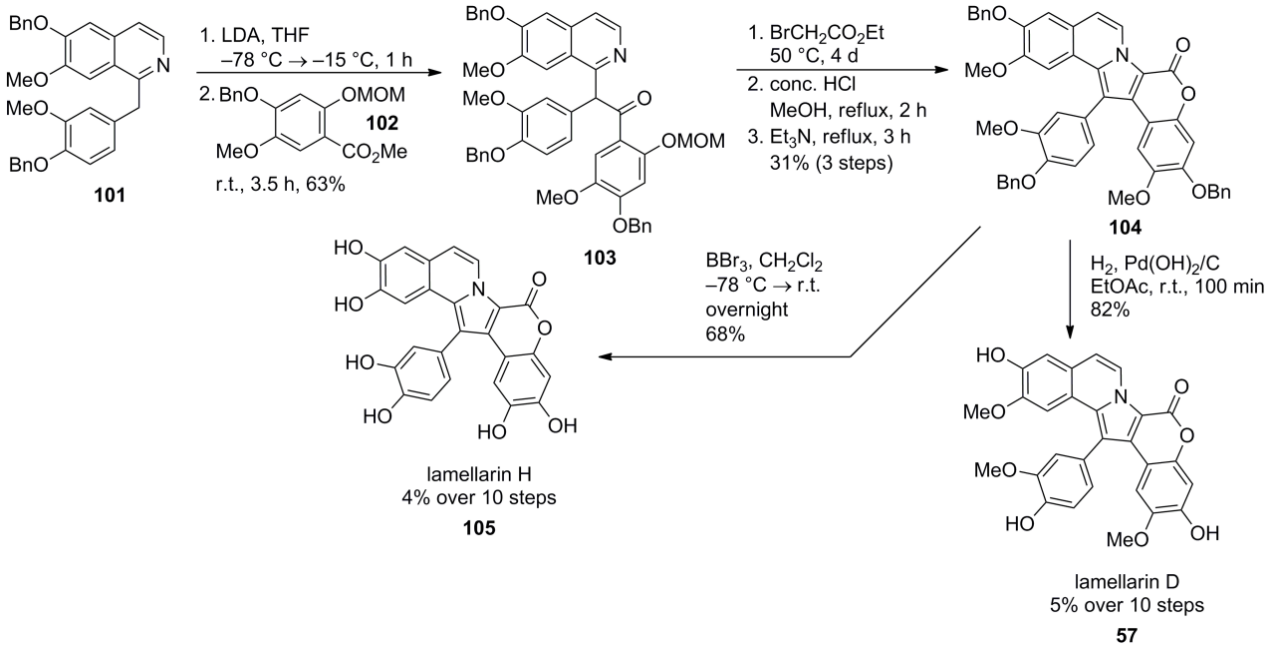
Synthesis of lamellarins D and H by Iwao *et al*. [82].

After benzylic deprotonation with LDA, **101** was reacted with benzoate **102** and furnished ketone **103** which was subsequently N*-*alkylated with ethyl bromoacetate followed by closure of the pyrrole ring. Acidic removal of the MOM-group and base promoted lactonization provided the protected type Ib-lamellarin **104**. The final deprotection was achieved by hydrogenolysis to provide lamellarin D (**57**) in 5% over 10 steps. Alternatively, complete O*-*deprotection was affected by BBr_3_ providing lamellarin H (**105**) in 4% over 10 steps from benzyl-protected isovanillin (Scheme 16).

### 4.2. Guitián 2001 [83]

In the approach of Guitián, the pyrrole core was constructed in a [3+2]-cycloaddition of nitrone **110**, obtained by a reduction/oxidation sequence from dihydropapaverine derivative **109**, to arylpropiolic acid ester **108**. The alkyne component **107** was synthesized starting from *O*-isopropylisovanillin (**106**) by iodination and Sonogashira coupling with TMS-acetylene. The latter compound was subjected to Baeyer-Villiger oxidation followed by hydrolysis of the resulting formate and protection as an isopropyl ether. Finally, the lithiation of the alkyne deprotected in the oxidation step and carboxylation with ethyl chloroformate provided arylpropiolate **108**.

**Scheme 17:**
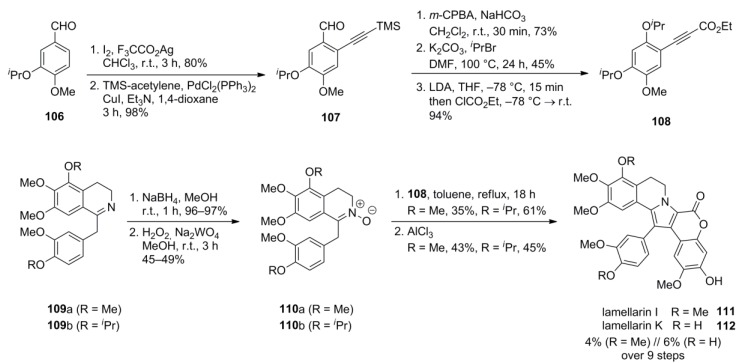
Synthesis of lamellarins I and K by Guitián *et al.* [83].

Dihydropapaverine derivative **109** was transformed into nitrone **110** by reduction with sodium borohydride followed by oxidation with disodium tungstate. [3+2]-Cycloaddition to alkyne **108** afforded the protected lamellarin**s**. Cleavage of the isopropyl ethers with AlCl_3_ provided lamellarins I (**111**) and K (**112**) in 4% and 6% over nine steps, respectively (Scheme 17).

### 4.3. Ruchirawat 2001 [84]

**Scheme 18:**
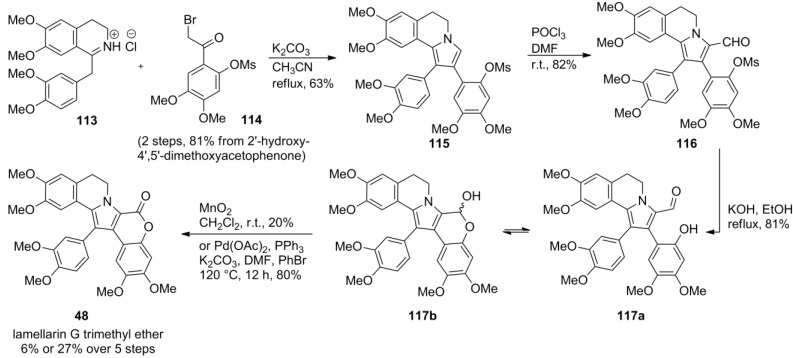
Synthesis of lamellarin G trimethyl ether by Ruchirawat *et al.* [84].

The cyclocondensation of commercially available dihydropapaverine hydrochloride **113** with phenacyl bromide **114** was utilized by Ruchirawat and co-workers in their key step to construct almost the entire lamellarin framework with the exception of the D-ring in a single operation. Vilsmeier formylation of the free position in the pyrrole ring followed by basic deprotection and manganese (IV) oxidation of the cyclic hemiacetal **117b** or, alternatively, palladium-catalyzed oxidation with bromobenzene afforded lamellarin G trimethyl ether (**48**) in 7% or 27% over five steps starting from 2′-hydroxy-4′,5′-dimethoxyacetophenone (Scheme 18).

### 4.4. Ruchirawat 2004 (lamellarin K&L) [85]-2006 (28 different lamellarins) [86]-Opatz 2008 (lamellarin G trimethyl ether & lamellarin U) [87]

The Ruchirawat-group envisioned that the pentasubstituted pyrrole moiety could also arise from a cyclocondensation of a 1-benzyl-3,4-dihydroisoquinoline (**122**), serving as an enamine precursor, with an α-nitrocinnamate (**123**) in a Grob pyrrole synthesis. Both components could easily be prepared from simple starting materials, allowing a highly modular and straightforward access to the lamellarins.

**Scheme 19:**
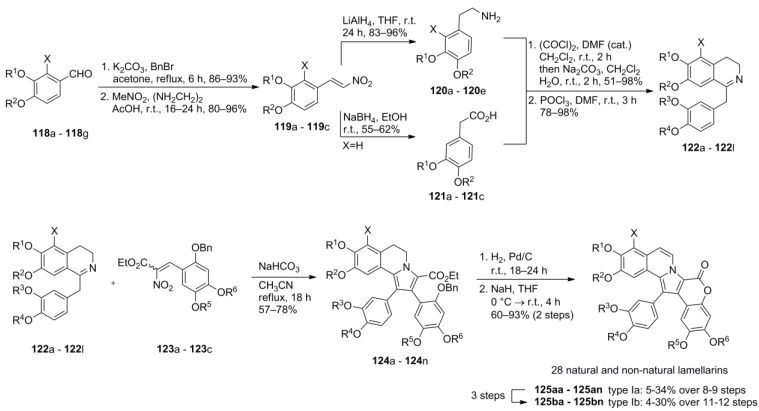
Synthesis of natural and non-natural occurring lamellarins by Ruchirawat *et al.* [86].

At first, Ruchirawat and co-workers reported the synthesis of lamellarin L and K employing the aforementioned strategy. Later on, the authors reported the hitherto most comprehensive synthetic work including the synthesis of 28 natural and non-natural lamellarins (**125**). The required dihydroisoquinolines **122** were prepared in an eight step synthesis as the longest sequence starting from vanillin, isovanillin and 2,3,4-trimethoxybenzaldehyde by O*-*benzylation followed by nitroaldol condensation, LiAlH_4_- or NaBH_4_-reduction to the 2-arylethylamines **120**. Treatment of these intermediates with sodium nitrite produced arylacetic acids **121**, the other coupling partners for preparing the intermediates **122**. This was achieved by conversion into acid chlorides and subsequent amide formation with amines **120** followed by Bischler-Napieralski cyclization. The α-nitrocinnamates **123** were in turn prepared starting from protected aromatic aldehydes in a five step sequence including Baeyer-Villiger oxidation, basic hydrolysis, repeated benzylation, Vilsmeier formylation and final Knoevenagel condensation with ethyl nitroacetate. The Grob cyclization smoothly provided the highly functionalized pyrroles **124** which were transformed into the lamellarins with a saturated B subunit by hydrogenolysis and basic lactonization. An additional three step protocol comprising O*-*acetylation, DDQ oxidation to establish the 5,6-double bond and subsequent deacetylation was developed for the transformation of type Ia- into type Ib-lamellarins. Unfortunately, this strategy leads to inferior yields and requires more difficult workup procedures compared to the strategy of Iwao and co-workers [73]. Nonetheless, Ruchirawat’s approach permitted the straightforward synthesis of more than two dozen lamellarins, unifying an inexpensive and a labor saving method, as only two of the steps require a chromatographic purification of the reaction products (Scheme 19).

**Scheme 20:**
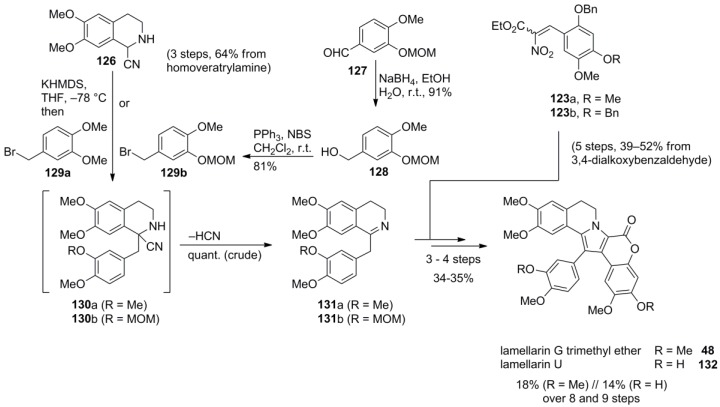
Synthesis of lamellarin G trimethyl ether and lamellarin U by Opatz *et al.* [87].

In 2008, Opatz and co-workers reported a modified version of Ruchirawat’s synthesis which emphasized the advantages of using tetrahydroisoquinoline-1-carbonitrile **126** as a key building block for the otherwise analogous route. Acid-sensitive groups could be included in the dihydroisoquinoline moiety as the harsh conditions of the Bischler-Napieralski reaction were avoided at that stage. The pivotal α-aminonitrile **126 **was prepared from homoveratrylamine in three steps and 64% yield (later improved to 84%, *vide infra*). Its C-benzylation after deprotonation with KHMDS directly furnished the dihydroisoquinolines **131** which were then reacted with α-nitrocinnamates **123** to produce lamellarin U (**132**) and lamellarin G trimethyl ether (**48**) in 14% and 18% overall yield over nine and eight steps, respectively (Scheme 20).

## 5. Synthesis of type-I Lamellarins—Miscellaneous Approaches

### 5.1. Banwell 1997 [88]//Faulkner 2002 [89]//Alvarez 2003 [90]

**Scheme 21:**
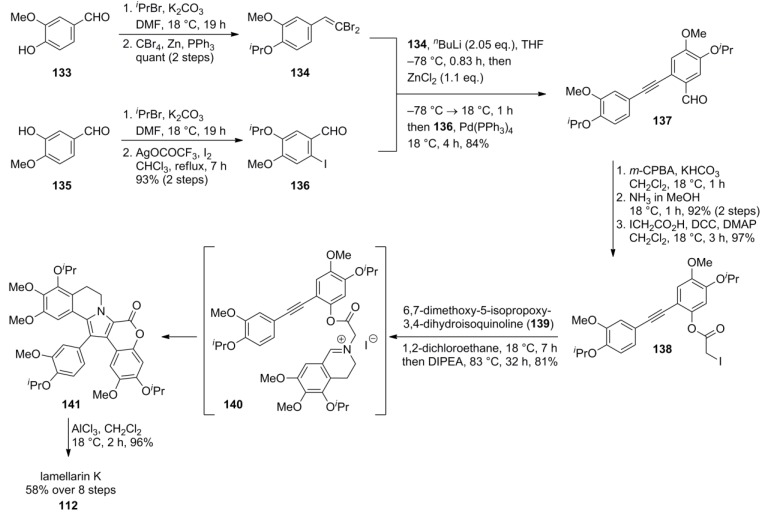
Synthesis of lamellarin K by Banwell *et al.* [88].

Banwell and co-workers were first to report a convergent total synthesis of type I-lamellarins via an intramolecular [3+2] cycloaddition of an azomethine ylide derived from the tolane-linked dihydroisoquinolinium salt **140**. The pivotal tolane **137** was synthesized from dibromostyrene **134** and iodide **136**, both accessed from vanillin (two steps—quantitative yield) and isovanillin (two steps—93%), respectively. Styrene **134** formed the lithium acetylide upon treatment with *^n^*BuLi which was transmetallated into the alkynylzinc chloride to provide tolane **137** after Negishi cross-coupling with iodide **136**. Baeyer-Villiger oxidation, basic hydrolysis and DCC-mediated condensation with iodoacetic acid provided the ester **138** which readily formed the dihydroisoquinolinium salt **140**. Upon deprotonation, the 1,3-dipole underwent cyclization to triisopropyllamellarin K (**141**). Final cleavage with AlCl_3_ gave lamellarin K (**112**) in 58% over eight steps (Scheme 21). Along the same lines, the Banwell group reported the synthesis of lamellarin T (41%, six steps), U (**132**, 45%, six steps), and W (42%, seven steps), all starting from 3-isopropoxy-4-methoxybenzaldeyde, in 2012 [91].

**Scheme 22:**
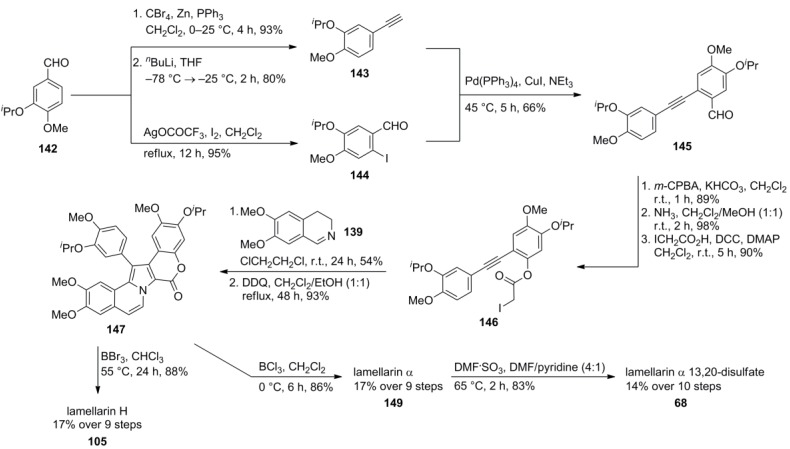
Synthesis of lamellarins H, α and α 13,20-disulfate by Faulkner *et al.* [89].

Faulkner and co-workers modified Banwell’s method by introducing the 5,6-double bond at a later stage, *i.e.*, after the pyrrole forming [3+2] cycloaddition [88] Lamellarin α (**149**) and lamellarin H (**105**) were each synthesized in 17% overall yield, starting from the isopropyl ether of isovanillin (**142**). Treatment of lamellarin α with DMF·SO_3_ in a mixture of DMF/pyridine (4:1) provided lamellarin α 13,20-disulfate (**68**) in 83% yield. However, attempts to introduce a single sulfate group were unsuccessful (Scheme 22).

**Scheme 23:**
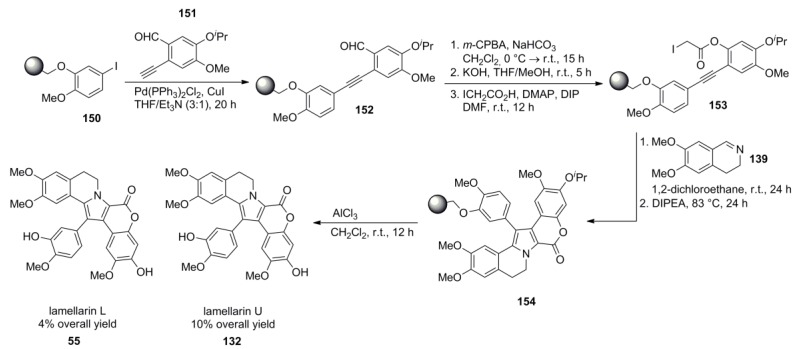
Solid-Phase-Synthesis of lamellarin U by Álvarez *et al.* [90].

Álvarez and co-workers developed a solid-phase supported version of Banwell’s route [88] and were able to synthesize lamellarin U (**132**) in 10% overall yield. Iodophenol **151** was linked to hydroxymethyl polystyrene resin by a Mitsunobu reaction and was subjected to Sonogashira cross‑coupling with acetylene **152**. Lamellarin L was also obtained by an unexpected demethylation in the cleavage step and was isolated in 4% yield (Scheme 23).

### 5.2. Steglich 1997 (lamellarin G trimethyl ether) [92]-2000 (lamellarin L) [77]-2006 (lamellarin G & K) [93]-2009 Guptón (Steglich Synthon) [94]

**Scheme 24:**
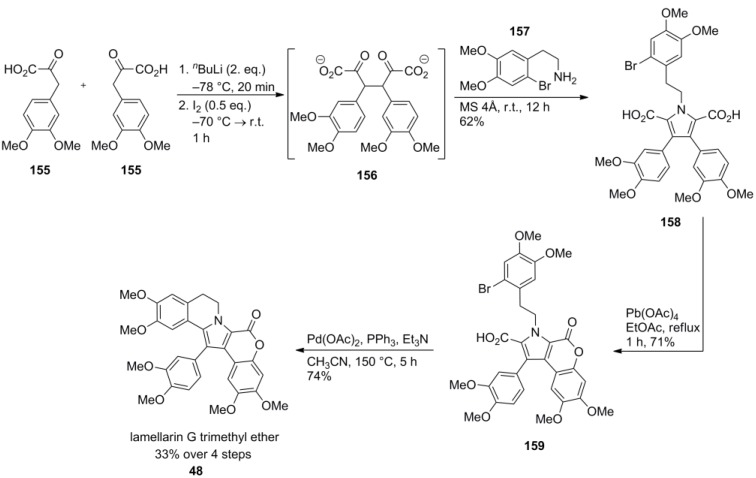
Synthesis of lamellarin G trimethyl ether by Steglich *et al.* [92].

Steglich and co-workers reported the very first synthesis of lamellarin G trimethyl ether (**48**) in a short, elegant and biomimetic fashion. Thus, a symmetrical pyrrole-2,5-dicarboxylic acid **158** containing rings A, E, and F was prepared by oxidative dimerization of 3-arylpyruvic acid **155** followed by Paal-Knorr cyclization with 2-arylethylamine **157**. Treatment of dicarboxylate **158** with lead (IV) acetate led to oxidative formation of the lactone ring. The B-ring was closed by an intramolecular palladium‑mediated Heck decarboxylation to afford lamellarin G trimethyl ether (**48**) in 33% over four steps. Until today, no shorter access to the core of the type I-lamellarins has been published (Scheme 24).

**Scheme 25:**
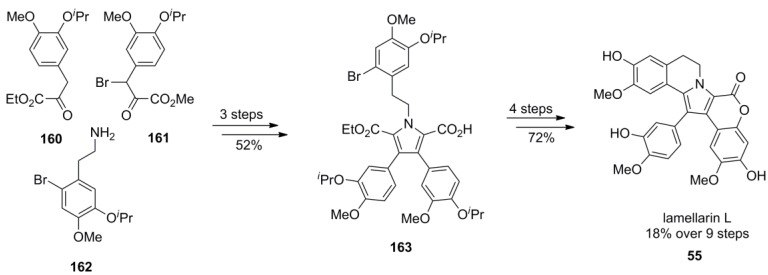
Synthesis of lamellarin L by Steglich *et al.* [77].

For the synthesis of lamellarin L (**55**), the carboxylate groups on the pyrrole ring were differentiated as a methyl and ethyl ester. Selective cleavage of the methyl ester applying a suspension of sodium cyanide in 1,3-dimethyl-3,4,5,6-tetrahydropyrimidin-2(1*H*)-one (DMPU), leaving the ethyl ester intact, allowed the discrimination of the attachment points for rings E and F. The required starting materials were all synthesized from the appropriate 3,4-disubstituted aromatic aldehydes which provided 2-arylethylamine **162**, ethyl arylpyruvate **160** and α-brominated methyl arylpyruvate **161** in 48%, 22% and 25% over three steps, respectively. Cyclization to key intermediate **163** was initiated by deprotonation of the ethyl ester with sodium hydride followed by C-alkylation with bromide **161** and condensation of *in situ* formed 1,4-diketone with arylethylamine **162**. After the above-mentioned differentiation of the ester units, monoacid **163** was subjected to lead (IV)-induced lactonization which exlusively formed the desired regioisomer. Saponification of the ethyl ester was effected with potassium hydroxide and the palladium-mediated decarboxylative cyclization afforded the isopropyl ether of **55**. Deprotection by AlCl_3_ gave lamellarin L (**55**) in 18% over nine steps (starting from *O*-isopropylisovanillin, Scheme 25).

**Scheme 26:**
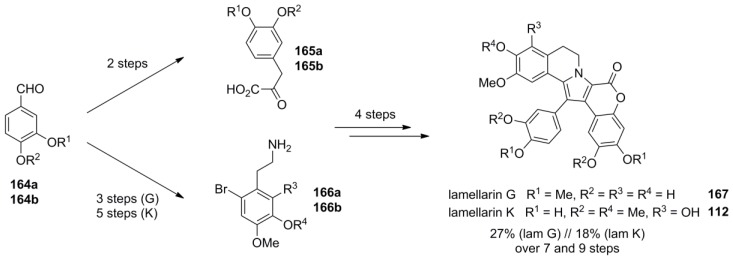
Synthesis of lamellarins G and K by Steglich *et al.* [93].

The same reaction sequence was applied to the synthesis of lamellarin G (**167**) and K (**112**) which were obtained in 27% over seven steps and 18% over nine steps, respectively. The lower yield of lamellarin K may be due to the increased sterical hindrance imposed by the additional *ortho*‑substituent (Scheme 26).

**Scheme 27:**
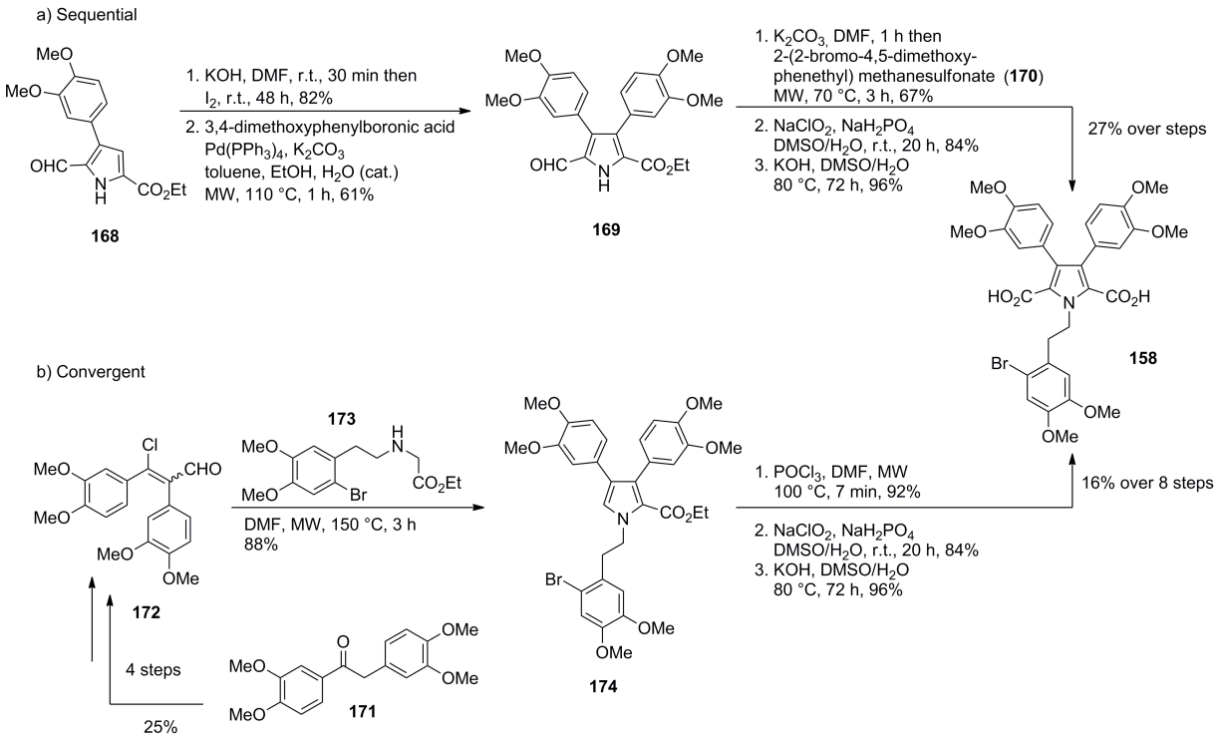
Synthesis of common intermediate **158** by Gupton *et al.* [94].

Later on, Gupton and co-workers reported two new methods for the synthesis of the intermediate **158** used by the Steglich group, one being sequential and the other one being convergent. Hence, the common intermediate **158** was prepared by the sequential route in 27% over five steps (starting from already prefunctionalized 2,3,5-trisubstituted pyrrole **168**) or by the convergent route in 16% over eight steps (starting from desoxyveratroin **171**, Scheme 27). Gupton also developed approaches to functionalized pyrroles suitable as precursors to type-I and type-II lamellarins and most recently published a second formal total synthesis of lamellarin G trimethyl ether [95,96].

### 5.3. Yadav 2009 [97]

**Scheme 28:**
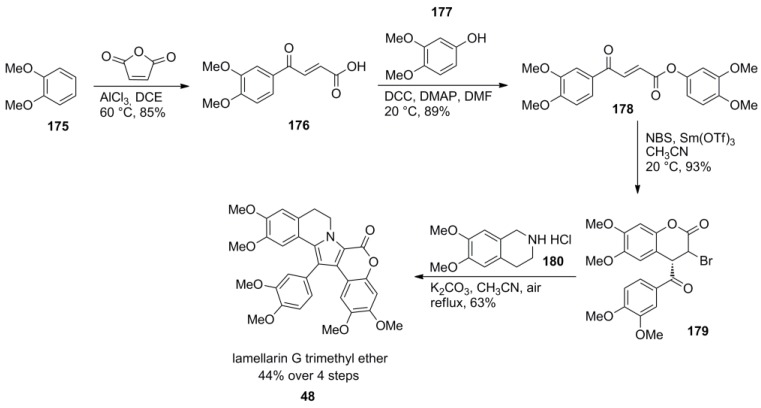
Synthesis of lamellarin G trimethyl ether by Yadav *et al.* [97].

An efficient, elegant, and very short access to lamellarin G trimethyl ether (**48**) was published by Yadav and co-workers. Starting with a Friedel-Crafts acylation of veratrole (**175**) with maleic anhydride to *trans*-acid **176**, esterification with 3,4-dimethoxyphenol (**177**) and intramolecular haloarylation furnished α-bromolactone **179**. A substitution reaction with 6,7-dimethoxy-1,2,3,4-tetrahydroisoquinoline (**180**) and aerobic oxidation/deprotonation provided the azomethine ylide which underwent a cyclocondensation to the target lamellarin **48** which was obtained in 44% over four steps. The synthesis represents the first example of an early construction of subunit E and lactone-subunit D before the construction of the pyrrole moiety (Scheme 28).

### 5.4. Jia 2011 [70]

The same biomimetic approach comprising three oxidative ring closures as used in their synthesis of lamellarin R (*vide supra*) was employed by Jia and co-workers for a concise route to lamellarin D and H.

**Scheme 29:**
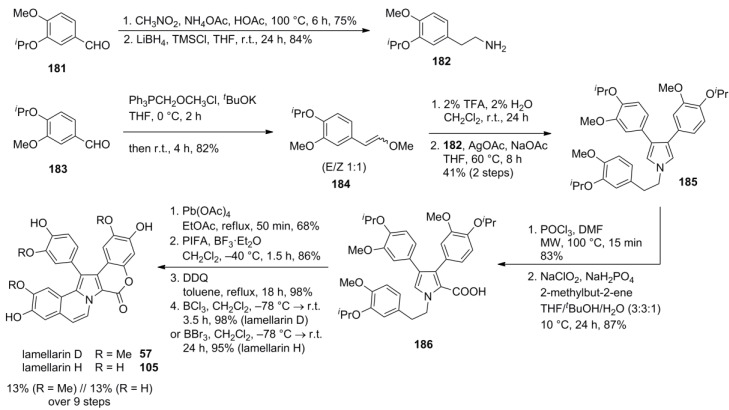
Synthesis of lamellarins D and H by Jia *et al.* [70].

Starting from *O*-isopropylvanillin (**183**) and *O*-isopropylisovanillin (**181**), O*-*protected 2-arylethylamine **182** and 3,4-dialkoxy-substituted phenylacetaldehyde **184** were prepared. AgOAc‑mediated oxidative coupling afforded pyrrole **185** in the key step. Vilsmeier-Haack formylation under standard conditions led to either decomposition or resulted in no conversion at all. However, microwave irradiation solved that problem and after various attempts, Pinnick/Lindgren oxidation was found to provide carboxylic acid, albeit in a low yield. Further optimization revealed that the ratio of the solvents dramatically affected the conversion and the outcome of the oxidation, indicating that THF/*^t^*BuOH/H_2_O (3:3:1) as the solvent system afforded carboxylic acid **186** in high yield. Oxidative lactonization according to Steglich and annulation according to Iwao followed by DDQ-mediated dehydrogenation of the B-ring provided lamellarin D (**55**) after BCl_3_-mediated deprotection in 13% over nine steps. Cleavage of all ether groups by the stronger Lewis acid BBr_3 _gave lamellarin H (**105**) in 13% over ten steps as well (Scheme 29).

### 5.5. Opatz 2013 [16]

**Scheme 30:**
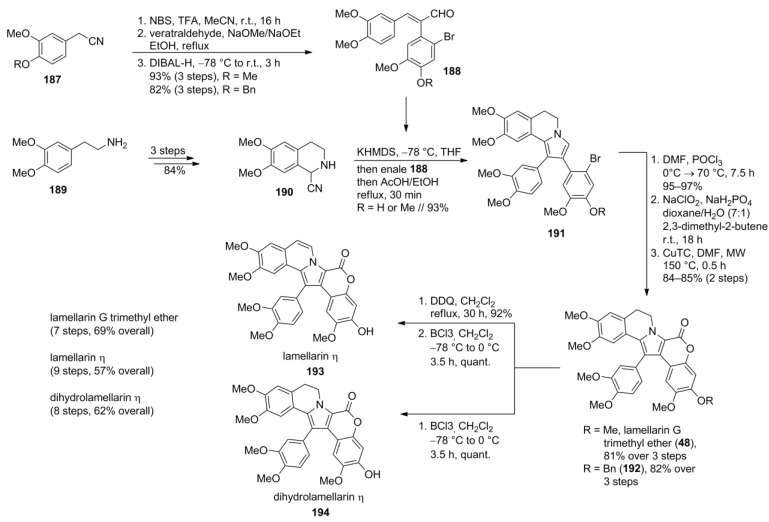
Synthesis of lamellarin G trimethyl ether, dihydrolamellarin η and lamellarin η by Opatz *et al.* [16].

The so far highest yielding and fully modular total synthesis of the type I-lamellarins was recently reported by Opatz and co-workers. In a first key step, an extended von Miller-Plöchl-type reaction of a quantitatively deprotonated α-aminonitrile **190** to α,β-unsaturated aldehydes **188** provided pyrrolo[2,1-a]isoquinolines **191** in high yield (both 93%). The Michael acceptors **188** as well as the bicyclic α‑aminonitrile **190** were readily prepared from vanillin and homoveratrylamine (**42**). Vilsmeier formylation followed by Pinnick/Lindgren oxidation provided pyrrole carboxylic acids which were used without purification as crude DMF solutions due to their instability which was observed by Jia and Ruchirawat as well [70,85]. In contrast to Jia’s protocol, 1,4-dioxane/H_2_O (7:1) was used as the solvent in the oxidation step. Ullmann-like lactone formation mediated by copper thiophene-2-carboxylate (CuTC) served as the second key step and completed the lamellarin core. Lamellarin G trimethyl ether (**48**) was obtained in 67% over seven steps, dihydrolamellarin η (**194**) and lamellarin η (**193**) were obtained in 62% (eight steps) and 57% (nine steps), respectively, after optional dehydrogenation (DDQ) and BCl_3_-promoted deprotection (Scheme 30).

### 5.6. Yamaguchi 2014 [98]

A concise synthesis of lamellarin C (**201**) and I (**111**) was recently presented by Yamaguchi and co‑workers. The first general β-selective C-H-arylation of pyrroles was combined with an intramolecular double oxidative biaryl coupling to generate the B- and the D-ring in a single operation.

**Scheme 31:**
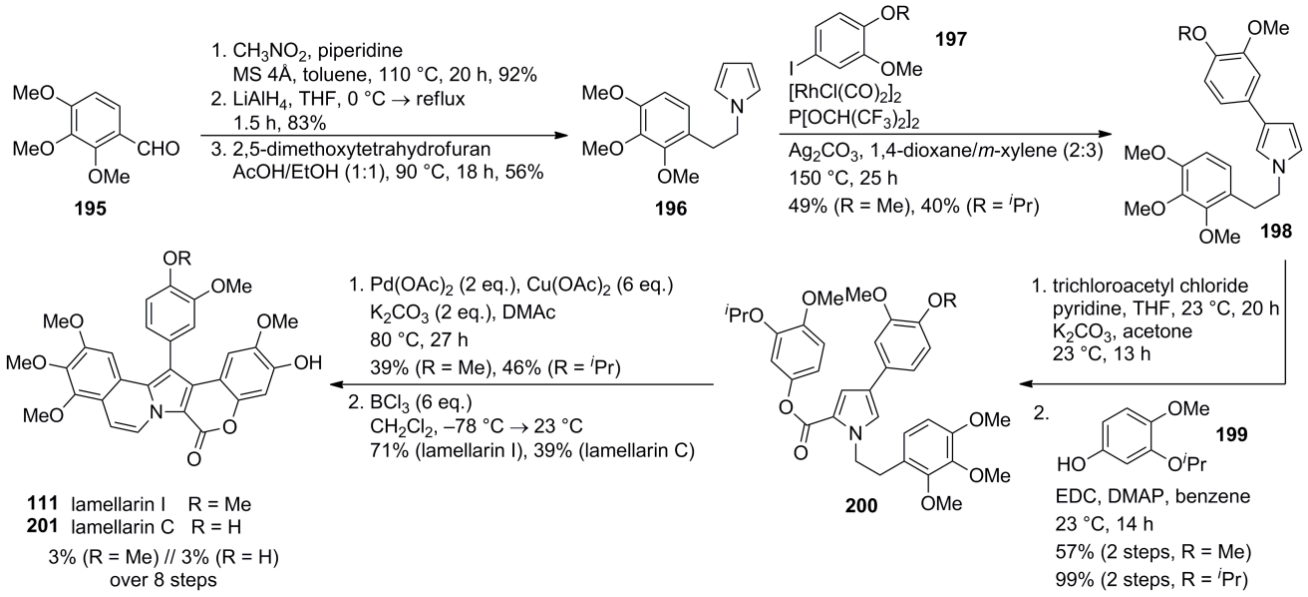
Synthesis of lamellarins C and I by Yamaguchi *et al.* [98].

Starting from 2,3,4-trimethoxybenzaldehyde (**195**), a sequence comprising nitroaldol condensation, LiAlH_4_ reduction and Paal-Knorr pyrrole synthesis was employed to obtain the N*-*substituted pyrrole **196**. Rhodium-catalyzed arylation with iodoarenes **197** occurred exclusively at C3 and was followed by treatment with trichloroacetyl chloride. Hydrolysis and esterification of the produced carboxylic acid provided phenolic ester **200**. Oxidation with palladium (II)-acetate (2 equiv.) and copper (II)-acetate (6 equiv.) furnished the pentacyclic ABCDE-framework after preparative thin layer chromatography (22 and 46% yield over 3 steps, respectively). The singly coupled side product could be isolated by gel permeability chromatography from the complex residual mixture. Global deprotection with BCl_3_ afforded lamellarins I (**111**) and C (**201**) in 3% over eight steps (Scheme 31).

## 6. Conclusions

Since their discovery in 1985, the lamellarin alkaloids have attracted the attention of scientists from the areas of biochemistry, synthetic organic chemistry, pharmaceutical and medicinal chemistry as well as biology, pharmacology, and medicine. Since 1995, more than 30 syntheses of the original lamellarins have been published. If related pyrrole-derived alkaloids such as lukianol A, the polycitrins A and B, the polycitones A and B, storniamide A, the didemnimide A–C and the ningalins A–D are also taken into account, this number is easily doubled. The synthetic approaches to the lamellarins are not limited to the naturally occurring representatives but also permit the preparation of analogues. Moreover, they allow substantial amounts of the lamellarins to be made which are required for investigations of their biological properties. Some of the syntheses rely on hidden symmetry in their target and are not suitable for the preparation of the majority of the other lamellarins.

On the other hand, modular strategies were reported which build up the lamellarin scaffold from commercial or readily available simple starting materials. A major class of syntheses employs a cyclocondensation or a cycloaddition reaction to establish the pyrrole core from precursors already carrying the alkoxylated benzene units later to become rings A, E, and F. In a second large class of syntheses, a simple pyrrole derivative is sequentially decorated with the required aryl moieties, mostly using halogenation/cross-coupling methodology.

The most prominent advantage of the cross-coupling based strategies is the possibility to generate a multitude of substitution patterns from libraries of differentially protected or O*-*methylated 3,4-dihydroxyphenylboronic acids in a combinatorial fashion. On the other hand, cyclocondensation or cycloaddition based strategies allow the construction of the entire lamellarin framework from prefunctionalized precursors under avoidance of yield-diminishing long linear reaction sequences. Thus, it is not surprising that the most efficient routes belong to the latter category. A comparison of the reported syntheses of the type-I lamellarins can be found in Table 1.

**Table 1:** Comparison of completed total syntheses of the type-I lamellarins.

Lead Author	Year	Lamellarin	Overall Yield	Linear Steps	Starting from
*Steglich*	1997	G trimethyl ether	33%	4	3-(3,4-Dimethoxyphenyl)pyruvic acid
*Banwell*	1997	K	58%	7	4-Isopropoxy-3-methoxybenzaldehyde
*Iwao*	1997	H	4%	10	3-Benzyloxy-4-methoxybenzaldehyde
		D	5%	10	3-Benzyloxy-4-methoxybenzaldehyde
*Steglich*	2000	L	18%	9	3-Isopropoxy-4-methoxybenzaldeyde
*Guitián*	2001	I	4%	9	3-Isopropoxy-4-methoxybenzaldeyde
		K	6%	9	3-Isopropoxy-4-methoxybenzaldeyde
*Ruchirawat*	2001	G trimethyl ether	27%	5	2‘-Hydroxy-4′,5′-dimethoxyacetophenone
*Faulkner*	2002	H	17%	9	3-Isopropoxy-4-methoxybenzaldehyde
		α	17%	9	3-Isopropoxy-4-methoxybenzaldehyde
		α 13,20-disulfate	14%	10	3-Isopropoxy-4-methoxybenzaldehyde
*Iwao*	2003	G trimethyl ether	12%	9	Homoveratrylamine
*Handy*	2004	G trimethyl ether	10%	8	Ethyl 4-bromopyrrole-2-carboxylate
*Álvarez*	2005	D	3%	16	3-Isopropoxy-4-methoxybenzaldehyde
*Iwao*	2006	N	16%	12	3-Isopropoxy-4-methoxybenzaldeyde
		D	18%	12	3-Isopropoxy-4-methoxybenzaldeyde
		L	19%	11	3-Isopropoxy-4-methoxybenzaldeyde
	2006	α 20-sulfate	24%	14	Homoveratrylamine
*Steglich*	2006	K	18%	9	4-Bromo-2,3-dimethoxybenzaldehyde
		G	27%	7	3-Isopropoxy-4-methoxybenzaldeyde
*Ruchirawat*	2006	I, C, T, F, K, E, dihydro‑η, G trimethyl ether, J, U, L, G, χ, Y ξ, B, W, ε, M, X, η, J‑DB, α, N, G‑DB, D, Y‑DB	5%–34% (saturated) 4%–30% (unsaturated)	8–9 (saturated) 11–12 (unsaturated)	3-Benzyloxy-4-methoxybenzaldehyde 4-Benzyloxy-3-methoxybenzaldehyde Veratraldehyde 2,3,4-Trimethoxybenzaldehyde 2,4-Dibenzyloxy-5-methoxybenzaldehyde 2,5-Dibenzyloxy-4-methoxybenzaldehyde 2-Benzyloxy-4,5-dimethoxybenzaldehyde
*Opatz*	2008	U	14%	9	3-Benzyloxy-4-methoxybenzaldehyde
		G trimethyl ether	18%	8	Veratraldehyde
*Yadav*	2009	G trimethyl ether	44%	4	Veratrole
*Iwao*	2010	α 20-sulfate	6%	15	3-Isopropoxy-4-methoxybenzaldeyde
		α 13-sulfate	4%	15	3-Isopropoxy-4-methoxybenzaldeyde
		α 13,20-disulfate	9%	14	3-Isopropoxy-4-methoxybenzaldeyde
*Banwell*	2011	G trimethyl ether	3%	10	*N*-Boc-pyrrole
		S	7%	11	3,4-Diisopropoxybenzaldehyde
*Jia*	2011	D	13%	9	4-Isopropoxy-3-methoxybenzaldehyde
		H	13%	9	4-Isopropoxy-3-methoxybenzaldehyde
*Banwell*	2012	T	41%	6	3-Isopropoxy-4-methoxybenzaldeyde
		W	42%	7	3-Isopropoxy-4-methoxybenzaldeyde
		U	45%	6	3-Isopropoxy-4-methoxybenzaldeyde
*Opatz*	2013	η	57%	9	4-Benzyloxy-3-methoxyphenylacetonitrile
		dihydro-η	62%	8	4-Benzyloxy-3-methoxyphenylacetonitrile
		G trimethyl ether	69%	7	3,4-Dimethoxyphenylacetonitrile
*Iwao*	2014	L	30%	13	*N*-Boc-2,5-dibromopyrrole
		N	42%	11	*N*-Boc-2,5-dibromopyrrole
*Yamaguchi*	2014	C	3%	8	2,3,4-Trimethoxybenzaldehyde
		I	3%	8	2,3,4-Trimethoxybenzaldehyde

The steps in the longest linear sequence were counted from the O-protected building blocks as the majority of the authors used this definition.

In the history of lamellarin synthesis, around 20 more or less different strategies have been devised until today. They exhibit a significant span of overall yields as well as the numbers of steps in the longest linear sequence but clearly, a decision on the suitability of an approach for solving a given synthetic problem also has to take into account the availability and price of the starting materials as well as the catalysts and reagents involved, let alone ecological issues which may become key factors in large-scale synthesis.

Due to the considerable attractivity of the lamellarin alkaloids, both from a biomedical and from a synthetic perspective, new approaches will continue to be developed. They will contribute to a better understanding of the chemistry as well as the biological activity of this compound class and may allow the rational design and preparation of more potent and/or more selective derivatives for prospective medicinal and pharmacological research.
